# LINE-1 couples EMT programming with acquisition of oncogenic phenotypes in human bronchial epithelial cells

**DOI:** 10.18632/oncotarget.21953

**Published:** 2017-10-23

**Authors:** Elsa M. Reyes-Reyes, Ivan Aispuro, Marco A. Tavera-Garcia, Matthew Field, Sara Moore, Irma Ramos, Kenneth S. Ramos

**Affiliations:** ^1^ University of Arizona College of Medicine, Division of Pulmonary, Allergy, Critical Care, and Sleep Medicine, Tucson, AZ, USA; ^2^ University of Arizona Cancer Center, Tucson, AZ, USA; ^3^ Center for Applied Genetics and Genomic Medicine, University of Arizona Health Sciences, Tucson, AZ, USA

**Keywords:** LINE-1, EMT programming, oncogenesis

## Abstract

Although several lines of evidence have established the central role of epithelial-to-mesenchymal-transition (EMT) in malignant progression of non-small cell lung cancers (NSCLCs), the molecular events connecting EMT to malignancy remain poorly understood. This study presents evidence that Long Interspersed Nuclear Element-1 (LINE-1) retrotransposon couples EMT programming with malignancy in human bronchial epithelial cells (BEAS-2B). This conclusion is supported by studies showing that: 1) activation of EMT programming by TGF-β1 increases LINE-1 mRNAs and protein; 2) the lung carcinogen benzo(a)pyrene coregulates TGF-β1 and LINE-1 mRNAs, with LINE-1 positioned downstream of TGF-β1 signaling; and, 3) forced expression of LINE-1 in BEAS-2B cells recapitulates EMT programming and induces malignant phenotypes and tumorigenesis *in vivo*. These findings identify a TGFβ1-LINE-1 axis as a critical effector pathway that can be targeted for the development of precision therapies during malignant progression of intractable NSCLCs.

## INTRODUCTION

Non–small cell lung cancers (NSCLCs) are the leading cause of cancer-related mortality and economic burden in the United States [1, 2]. Most patients present with locally advanced or metastatic disease, and current treatment modalities generally exhibit low response rates [3]. NSCLCs include adenocarcinomas, squamous cell carcinomas and large cell carcinomas, with several less common subtypes, namely, adeno-squamous carcinomas and sarcomatoid carcinomas, also included in the classification. During the past decade, the adoption of precision medicine approaches has significantly advanced lung cancer diagnosis and treatment, with new clinical management guidelines taking advantage of combined histologic and genetic characterization of tumors to implement targeted treatments. For the small subset of patients diagnosed with advanced lung adenocarcinoma carrying epidermal growth factor receptor (EGFR) mutations or anaplastic lymphoma kinase (ALK) rearrangements, several options including the tyrosine kinase inhibitors, erlotinib and gefitinib, are now available [4]. Likewise, an antibody against programmed death-ligand (PDL) was recently approved for the treatment of patients with advanced lung squamous cell carcinoma with PDL overexpression [5]. Despite tremendous progress, much remains to be learned about the primary molecular drivers in NSCLCs, the identity of additional targets for precise therapeutic interventions, and the mechanisms responsible for acquired drug resistance and disease relapse after surgery.

Several lines of evidence have established the profound influence of epithelial-to-mesenchymal transition (EMT) on NSCLC progression, metastasis and drug resistance [6, 7]. Of relevance is the observation that EMT programming is linked to migration of tumor cells into the circulation (7), and resistance to tyrosine kinase inhibitors [8–10]. During EMT, epithelial cells lose their polarity and cell-to-cell contacts to acquire migratory and invasive properties [7]. EMT programming has been loosely defined by the loss of cell–cell adhesion molecules (e.g. E-cadherin and ZO-1), down-regulation of epithelial differentiation markers (e.g. cytokeratins and claudins), and transcriptional induction of mesenchymal markers (e.g. vimentin, fibronectin and N-cadherin). Therefore, identification of the signaling pathways that regulate EMT programming can help to better define the molecular basis of cancer progression and identify novel therapeutic targets. The switch in genetic programming seen during EMT involves several key transcription factors including SNAIL, zinc-finger E-box binding (ZEB) and basic helix-loop-helix transcription factors, that regulate gene expression and set in motion a cascade of events that mediate the dissolution of cell junctions, cytoskeletal changes and increased migratory activity [7].

Members of the TGFβ family of proteins have been identified as inducers of EMT [11]. Of particular interest is the ability of TGF-β1 to induce invasive phenotypes in cancer cells [11]. TGF-β1 signaling is effected through a receptor complex that includes Type-I and Type-II transmembrane receptors with serine/threonine kinase activity. Type-II receptors phosphorylate type-I components to activate SMAD and non-SMAD pathways, such as p38MAPK, ERK, JNK, PI3K, and NF-κB [12]. In the case of SMAD signaling, receptor-activated SMAD2 and SMAD3 combine with SMAD 4 to form complexes that translocate to the nucleus and interact directly with gene promoters, or with transcriptional regulators to activate or repress gene expression [13]. TGF-β1-induced EMT is blocked by dominant negative forms of TGFbRII or TGFbRI, or pharmacological inhibition of kinase activity in many cell types, indicating that the response is mediated through these receptors [7]. The complexity of these molecular interactions is best exemplified by the finding that while SMAD3 positively regulates EMT, SMAD2 exerts an antagonist effect [14]. TGF-β1 induces SNAIL1 through SMAD-3-dependent transcription [14], and SMAD3-SMAD4 complexes cooperate with SNAIL to negatively regulate E-cadherin and occluding [15]. In epithelial cells undergoing EMT, TGF-β1 activates AKT and PI3K to drive EMT [7]. Clearly, the dynamic processes responsible for EMT are characterized by intersecting molecular pathways that afford considerable heterogeneity to the EMT response.

While it has been recognized that epithelial cell transdifferentiation induces migratory behavior in cancer cells, the molecular events that link EMT programming to malignancy remain poorly understood. Evidence is presented here that the Long Interspersed Nuclear Element-1 (LINE-1) retrotransposon couples EMT programming with tumorigenesis in human bronchial epithelial cells (BEAS-2B). These findings provide important insights into the role of LINE-1 in lung cancer and identify a TGFβ1-LINE-1-EMT axis as a molecular effector pathway that can be targeted for optimized treatments that target poor responsiveness of NSCLCs.

## RESULTS

### EMT in human bronchial epithelial cells challenged with TGF-β1

**Figure 1:**
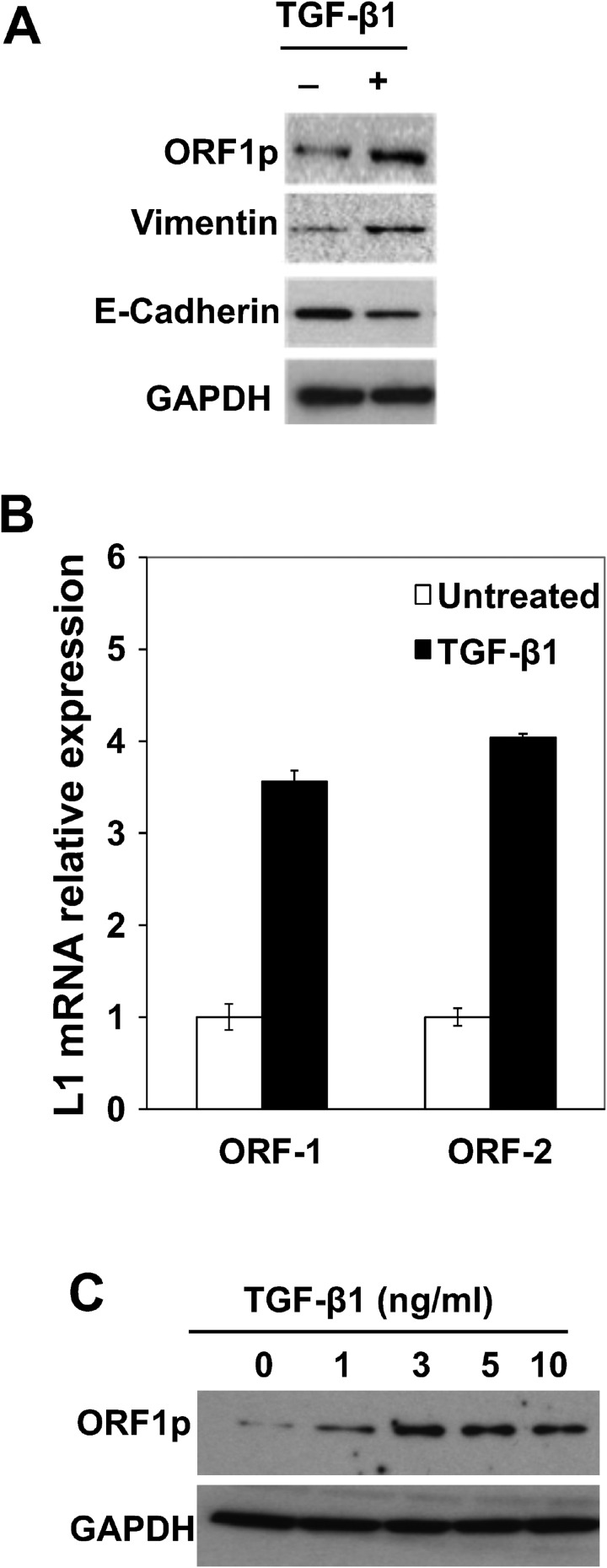
Activation of EMT Programming by TGF-β1 is Associated with LINE-1 Expression in Human Bronchial Epithelial Cells (**A**) BEAS-2B whole cell lysates isolated from cells stimulated with 3 ng/mL TGF-β1 for 48 hours or control were analyzed by immunoblotting using antibodies against LINE1 (L1) ORF1 protein (ORF1p), E-cadherin, vimentin or GAPDH. (**B**) Total RNA from untreated or treated with 3 ng/ml TGF-β1 for 8 hours, and 1 µg of RNA was subjected to cDNA synthesis. Samples were analyzed by RT-qPCR using specific primers for human L1 (ORF1 and ORF2). Expression levels are shown as the mean of triplicates with SEM relative to controls. (**C**) Whole cell lysates from cells stimulated with different concentrations of TGF-β1 for 48 hours or control were analyzed for expression of L1 ORF1p by immunoblotting. Data are representative of two or more independent experiments.

EMT has been linked to increased tumor migration in NSCLCs and to resistance to tyrosine kinase inhibitors [8–10]. To elucidate critical molecular events involved in this response, and its role in malignant transformation, BEAS-2B cells were challenged with 3 ng/mL TGF-β1 to activate EMT programming, and then processed for measurements of LINE-1 retrotransposon. BEAS-2B cells are immortalized, human bronchial epithelial cells that maintain a diploid genome in serial culture and are non-tumorigenic in nude mice. LINE-1 is a ubiquitous genetic element implicated in epithelial transdifferentiation during development and oncogenesis [16–18]. Its ability to modulate differentiation programs is linked to the production of ORF1 and ORF2, proteins involved in ribonuclear protein assembly, chromatin remodeling and altered gene expression [19]. Challenge of BEAS-2B cells with 3 ng/ml of TGF-β1 for 48 hours activated EMT programming, as evidenced by increased expression of mesenchymal vimentin and downregulation of epithelial cadherin (Figure 1A). This EMT response involved upregulation of LINE-1 ORF-1 and ORF-2 mRNAs (Figure 1B). Concentration-dependent increases in ORF1 protein were measured following treatment with TGF-β1, with peak induction seen at 3 ng/ml (Figure 1C). These results established the integrity of EMT programming in BEAS-2B cells in response to TGF-β1 and suggest LINE-1 retroelement is implicated in the transdifferentiation response of lung epithelial cells.

### Reactivation of LINE-1 by BaP is effected via canonical TGF-β1 signaling

**Figure 2:**
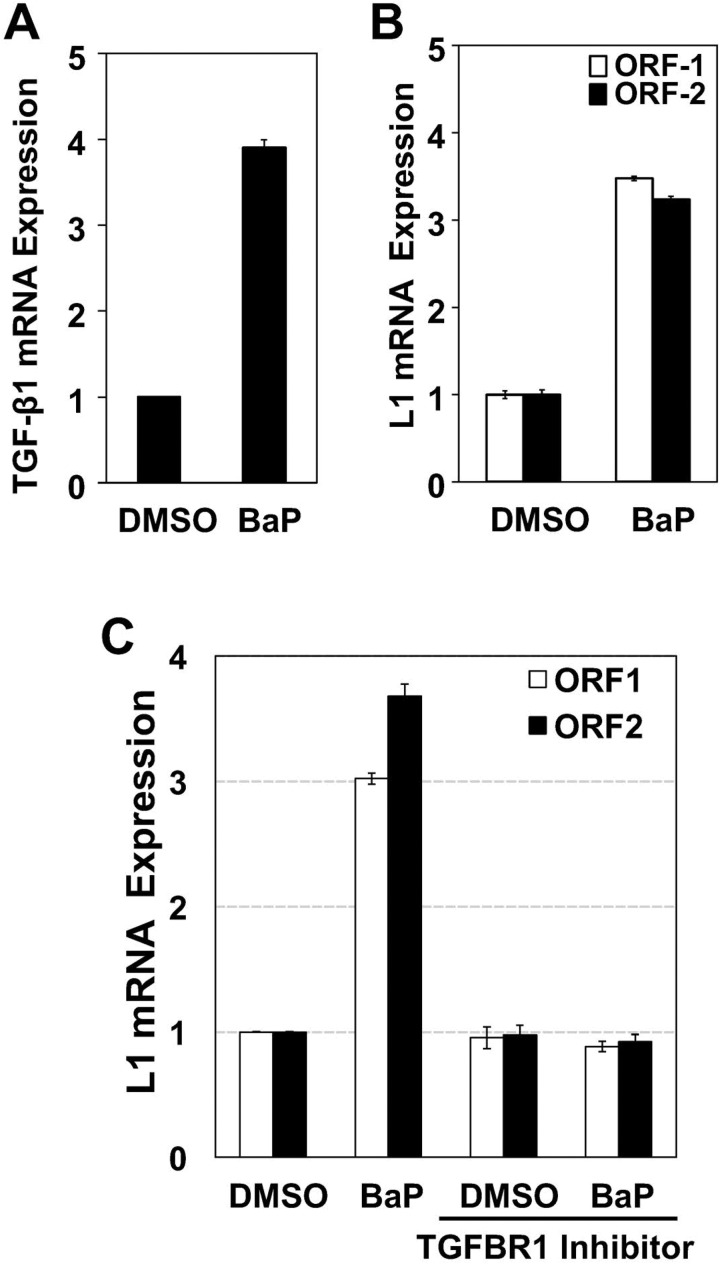

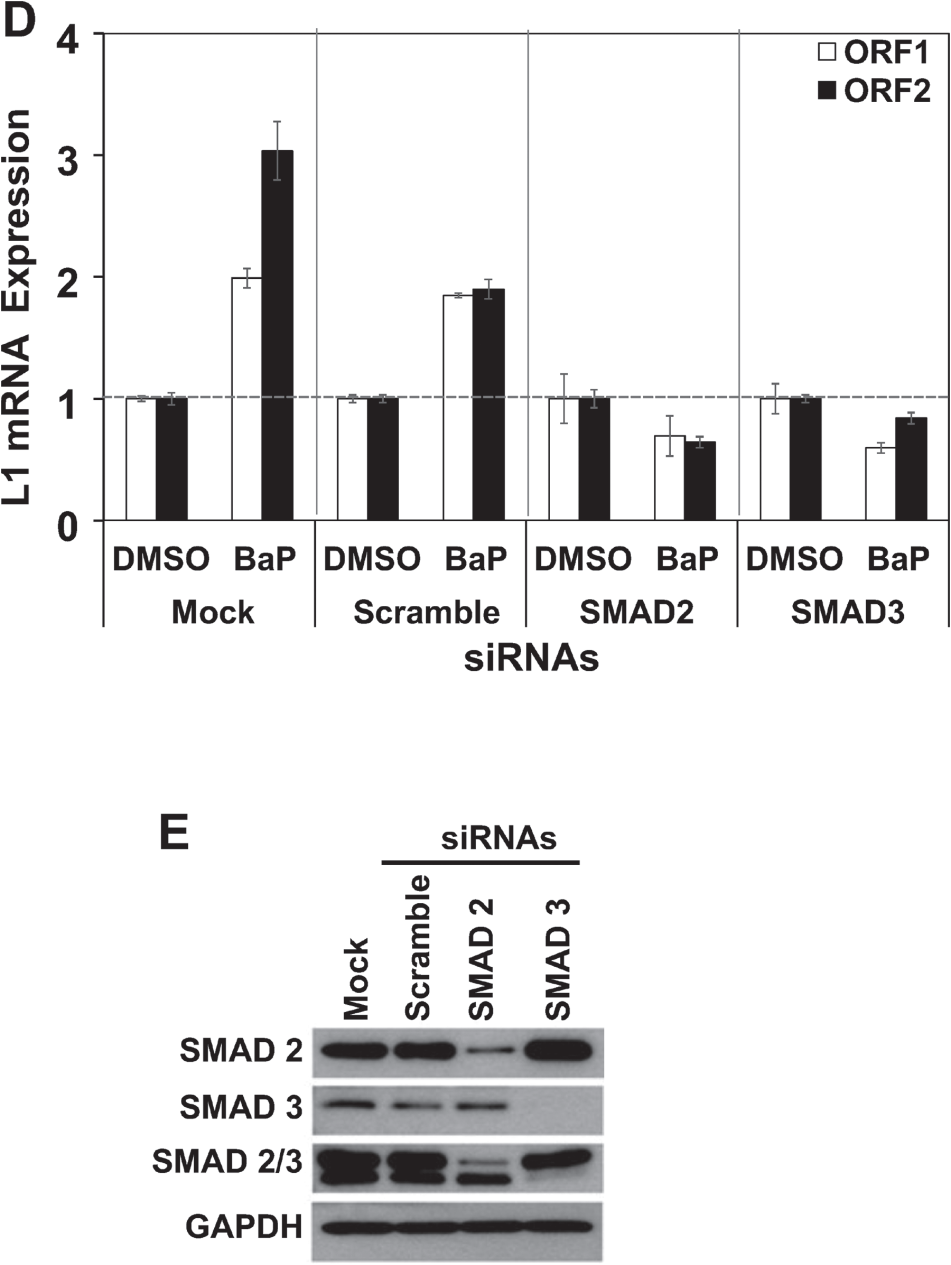
Reactivation of LINE-1 by BaP is Effected via Canonical TGF-β1 Signaling Total RNA was isolated from BEAS-2B cells treated with 0.5 uM BaP for 8 hours, and 1 µg of RNA subjected to cDNA synthesis. Samples were analyzed by RT-qPCR using specific primers for (**A**) human LINE-1 (ORF1 and ORF2) or (**B**) TGF-β1. (**C**) cells pre-treated with TGF-β1 receptor inhibitor (LY2157299) or vehicle (DMSO) for 30 min before BaP challenge or (**D**) transfected with target-specific siRNAs to SMAD2, SMAD3, or scramble siRNA or no siRNA (mock) controls. Expression levels are shown as the mean of triplicates with SEM relative to controls. (**E**) Whole cell extracts from transfected cells were analyzed by immunoblotting for SMAD2, SMAD3, SMAD2/3 or GAPDH antibodies (loading control) to confirm target knockdown. Data are representative of two or more independent experiments. Points represent mean of triple samples with SE.

The genome of lung carcinomas is one of the most frequently affected by LINE-1 insertions, with LINE-1-ORF1p expression restricted to high-grade lesions at advanced stages of tumorigenesis [20, 21]. These changes are consistent with the prominent roles played by both TGF-β1 and tobacco carcinogens in EMT programming and LINE-1 reactivation [17, 18]. To scrutinize patterns of molecular cross-regulation between TGF-β1 and LINE-1, BEAS-2B cells were challenged with the lung carcinogen BaP (0.5 µM) or vehicle (DMS0) followed by measurement of TGF-β1 and LINE-1 mRNAs by RT-qPCR. BaP increased mRNA levels of both TGF-β1 (Figure 2A) and LINE-1 ORF1 (Figure 2B). Peak induction for both targets was seen at 8 hours (not shown). Next, BEAS-2B cells were pretreated for 1 hour with 10 µM LY2157299, a TGFβR1 inhibitor being tested in human clinical trials [22, 23], or cells transfected with SMAD2 or SMAD3 siRNAS for target knockdown, before challenge with 0.5 µM BaP for 8 hours. LY2157299A completely blocked BaP-activated expression of LINE-1 ORF1 and ORF2 mRNAs (Figure 2C), specifying LINE-1 as a downstream target of TGF-β1 during the course of EMT programming. This interpretation was confirmed in experiments showing that genetic knockdown of downstream targets of TGF-β1 signaling, SMAD2 and SMAD3, also blocked LINE-1 inducibility (Figure 2D). The specificity of siRNAs was confirmed in Western blotting experiments where a > 70% reduction in the expression of SMAD2 and SMAD3 compared to controls (scramble siRNA or mock transfection) was observed (Figure 2E). Thus, regulation of LINE-1 by BaP in BEAS-2B cells lies downstream of TGF-β1 and couples to canonical TGF-β1 signaling.

**Figure 3:**
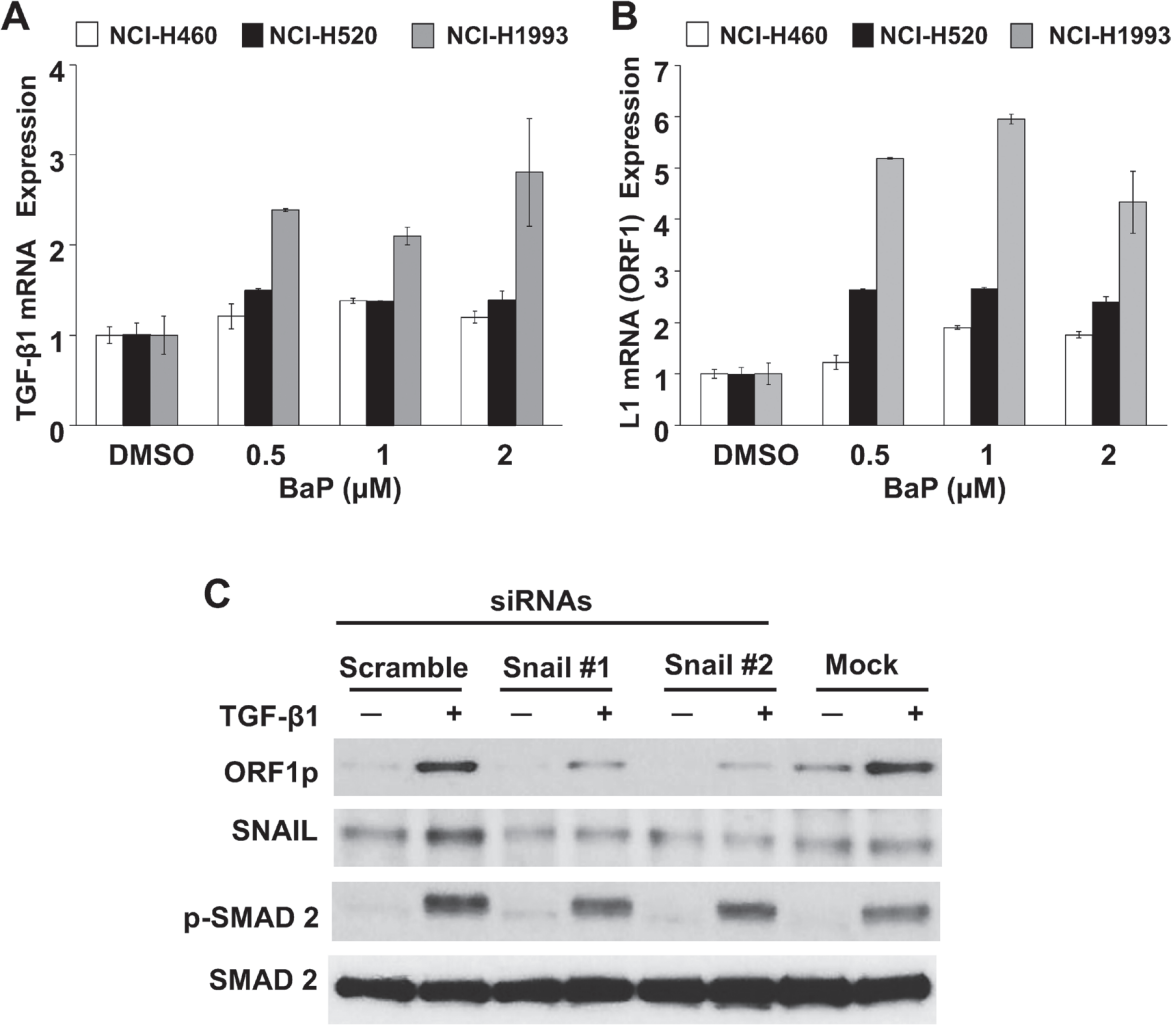
Specificity of TGF-β1/LINE-1 Interactions in Transformed Lung Epithelial Cell Lines NCI-H460, NCI-H520, or NCI-H1993 cell lines were challenged with BaP (0.5–2 uM) or 0.5% DMSO vehicle for 24 hours. Total RNA was isolated and 1 µg subjected to cDNA synthesis. Samples were analyzed by RT-PCR using specific primers for human TGF-β1 (**A**), LINE-1 (L1) ORF 1 (**B**) or GAPDH. Expression levels are presented relative to untreated cells. Each point represents the mean and SE of triplicate samples. The data are representative at two or more independent experiments. (**C**) Cells transfected with target-specific siRNAs to SNAIL or scramble siRNA or no siRNA (mock) were challenged with 3 ng/ml TGF-β1. Whole cell extracts were analyzed by immunoblotting for ORF1p, SNAIL, phospho(p)-SMAD2, or total SMAD2. Data are representative of two or more independent experiments.

To confirm the specificity of functional interactions between TGF-β1 and LINE-1, and to determine if the response was peculiar to the BEAS-2B cell line, a panel of NSCLC cell lines was examined (Figure 3). Reciprocal TGF-β1 and LINE-1 mRNA responses were observed in three different cancer lines (NCI-H460, NCI-H520 and NCI-H1993) (Figures 3A and 3B), with profiles comparable to those of naïve BEAS-2B cells. Further, genetic knockdown of the TGF-β1 target SNAIL blocked LINE-1 inducibility in NCI-H1993 cells challenged with 3 ng/mL TGF-β1 for 48 hours (Figure 3C). These findings confirm the integrity of the response across several epithelial cell lines, and indicate that functional interactions between TGF-β1 and LINE-1 are not restricted to the BEAS-2B cell line.

**Figure 4:**
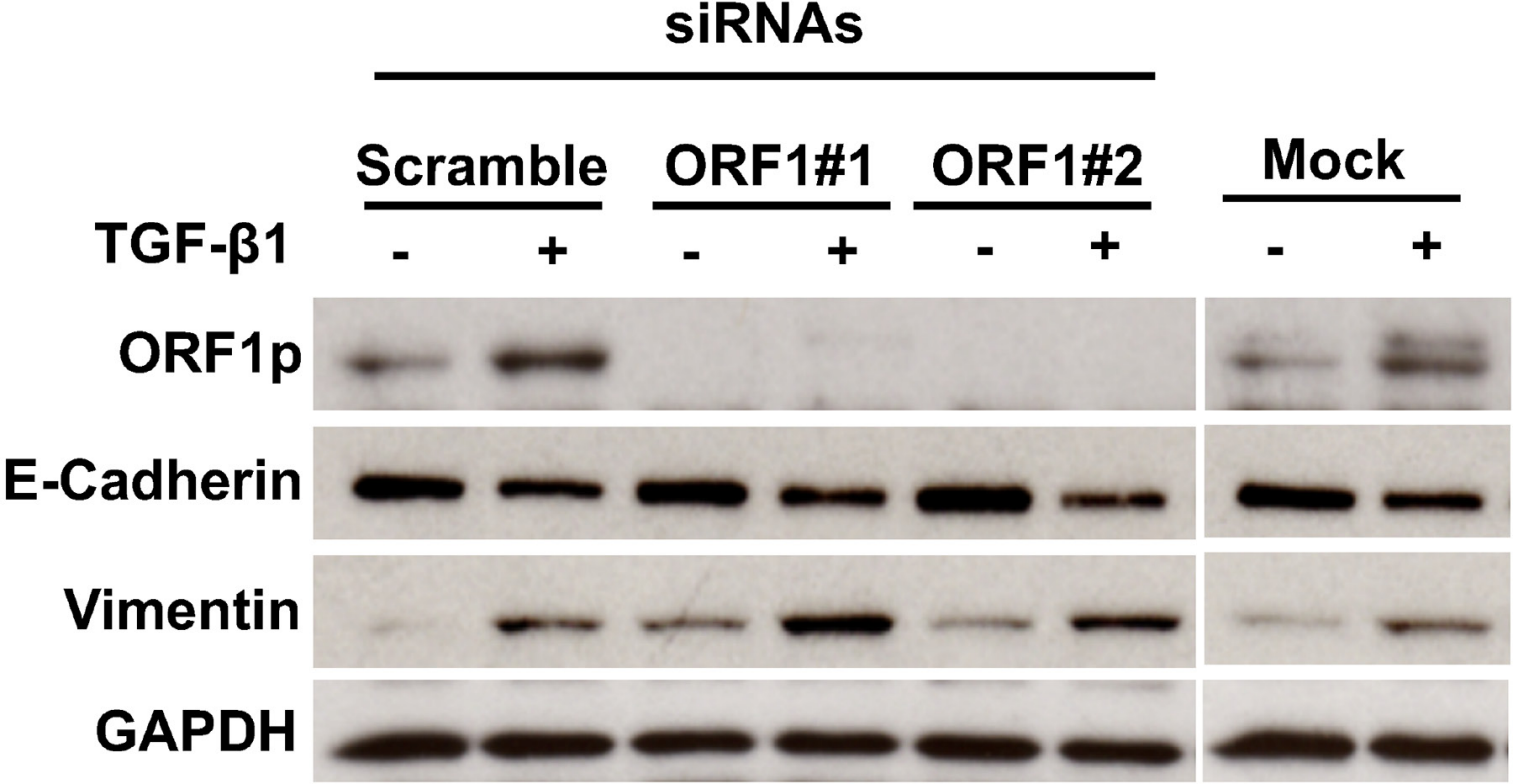
Impact of LINE-1 ORF-1 siRNAs on EMT Programming Cells were transfected with two unique target-specific siRNAs to LINE-1 targeting ORF1 regions or control siRNA (scramble) or no siRNA (mock), Forty-eight hours post-transfection, cells were challenged with 3 ng/ml TGF-β1 for an additional 48 hours or control. Whole cell lysates were analyzed by immunoblotting for ORF1p, E-Cadherin, vimentin or GAPDH.

In subsequent experiments, we tested the influence of siRNAs directed at LINE-1 ORF-1 on TGF-β1-induced EMT programming. Two ORF1-specific LINE-1 siRNAs and a control scramble siRNA or mock transfection were added for 48 hours before treatment with 3 ng/ml TGF-β1 for 48 hours. TGF-β1 induction of ORF1 protein was completely blocked in cells transfected with LINE-1 ORF1 siRNAs, but not in mock or scramble transfected cells (Figure 4). Specific knockdown of ORF1 protein did not reverse the EMT response to TGF-β1, as evidenced by intact responses for all transdifferentiation markers examined (Figure 4). These findings indicate that TGF-β1 signaling in bronchial epithelial cells during the course of EMT is effected through combinatorial pathways that extend beyond LINE-1 ORF1p. As such, expression of ORF1p alone is not sufficient to drive the EMT response mediated by TGF-β1.

### LINE-1 induces EMT phenotypes and resistance to anti-proliferative agents in human bronchial epithelial cells

**Figure 5:**
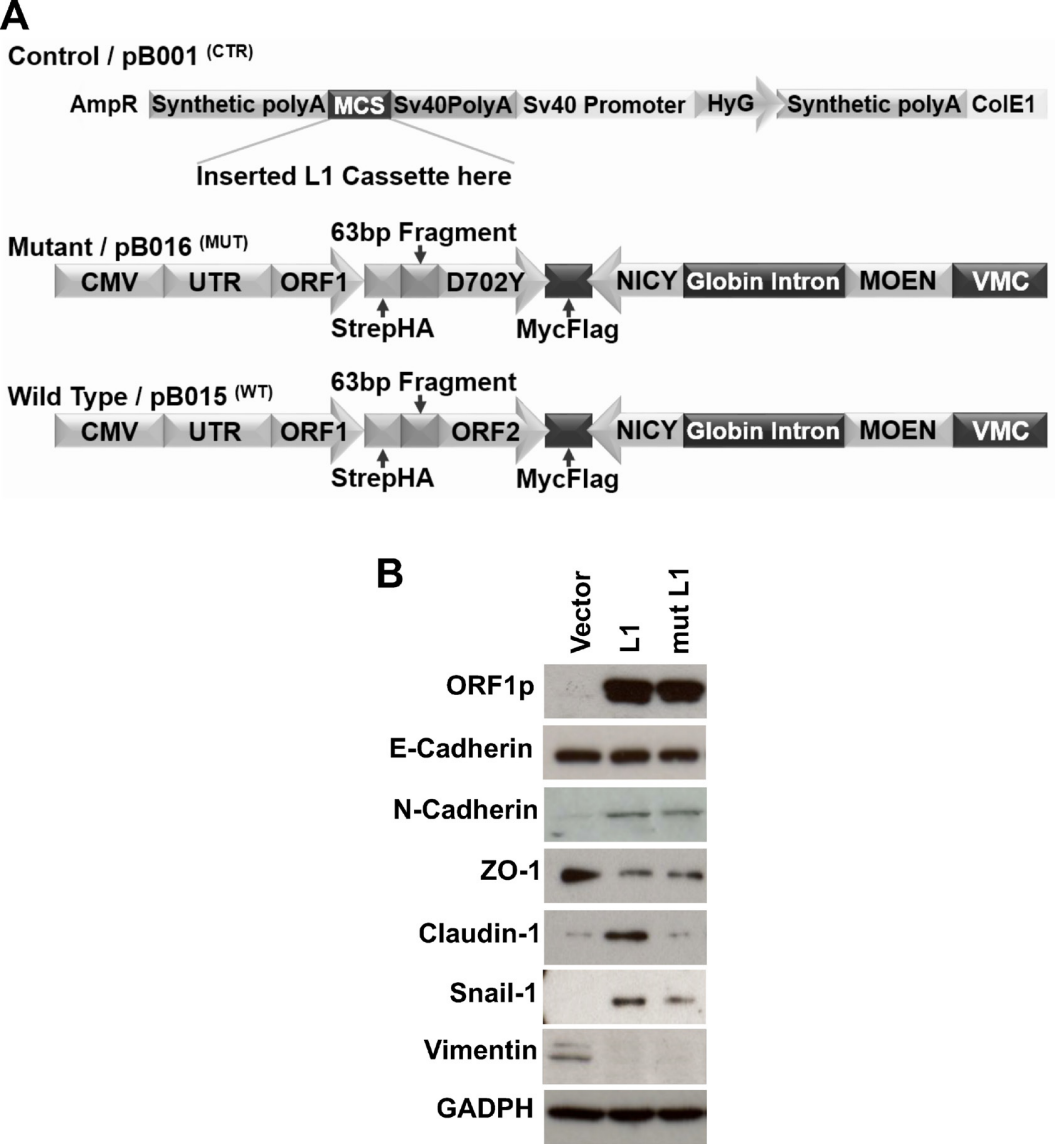
LINE-1 Induces EMT Phenotypes in Human Bronchial Epithelial Cells (**A**) Schematic representation of L1 expression vectors used to create stably transfected BEAS-2B cell clones. Vector controls lacked the L1 cassette. The mutant construct of LINE-1 carries a mutation in ORF2 (D702Y) lacking reverse transcriptase activity and rendered inactive for retrotransposition. The wild type LINE-1 construct contains retrotransposition competent ORF1 and ORF2 sequences. Both wild type and mutant vectors contain a neomycin cassette in antisense orientation to assay for retrotransposition activity. (**B**) Clones constitutively expressing wild type LINE-1 (L1), a mutant LINE-1 (mut L1), or empty vector control were generated by transfection using lipofectamine followed by selection of stably transfected clones with hygromycin. Whole cell lysates were analyzed by immunoblotting for L1 ORF1p, E-Cadherin, N-Cadherin, ZO1, Claudin-1, Snail-1, vimentin, or GAPDH. Data are representative two independent experiments using clones #5 (wild type L1) and #13 (mutant L1).

The putative roles of LINE-1 in driving cellular plasticity and neoplastic transformation of lung epithelial cells remain poorly defined. We previously showed that ectopic expression of LINE-1 induces EMT in hepatocarcinoma cells, a response that is independent of LINE-1 encoded reverse transcriptase activity [16, 24]. To determine whether LINE-1 expression modulates EMT programming in BEAS-2B cells, we stably transfected BEAS-2B with expression vectors for: 1) wild type LINE-1; 2) a mutant counterpart lacking reverse transcriptase activity and thus unable to retrotranspose (mutant L1); or 3) empty vector (Figure 5A). While the relative abundance of LINE-1 ORF1 protein across a large number of stably transfected clones was variable, clones #5 and #9 (wild type LINE-1) and clones #13 and #17 (mutant LINE-1) showed comparable protein expression levels (Supplementary Figure 1A). The proliferation rates of these four clonal cell populations, as measured by cell counts, were comparable (Supplementary Figure 1B). As such, clones # 5, 9, 13, and 17 were chosen for further phenotypic evaluation. BEAS-2B cells expressing wild type or mutant LINE-1 proteins exhibited increased expression of the mesenchymal markers N-Cadherin and SNAIL1, coupled with decreased expression of the epithelial marker ZO-1 compared to control cells (Figure 5B), while the expression of E-Cadherin and vimentin was unaffected. Claudin1 was selectively induced by wild type LINE-1, but not the mutant or empty vectors (Figure 5B). These data suggest that LINE-1 participates in transdifferentiation programming of polarized, non-malignant bronchial epithelial cells to induce EMT phenotypes. Further, the variable degrees of EMT programming in BEAS-2B cells implicate complex contributions by canonical and non-canonical TGF-β1 signaling pathways, as well as LINE-1 downstream of TGF-β1, in the EMT response.

**Figure 6:**
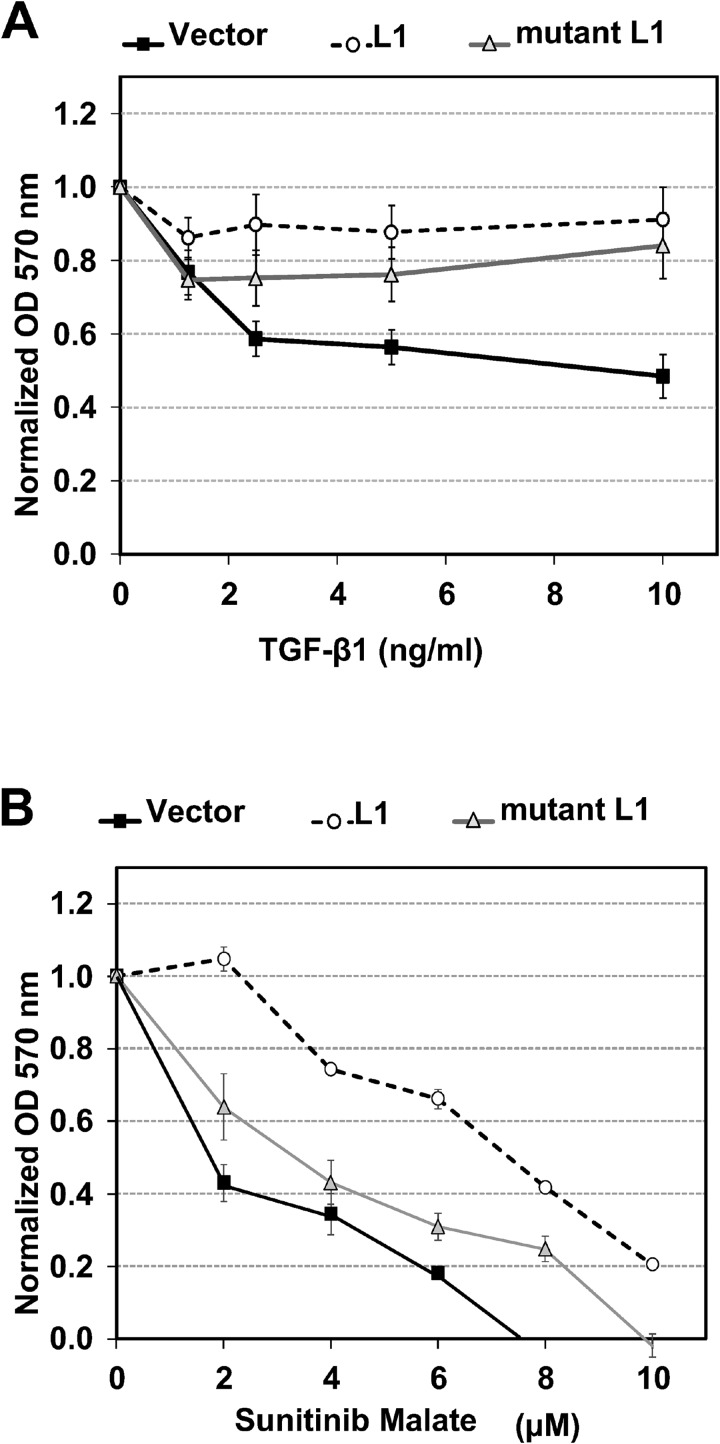

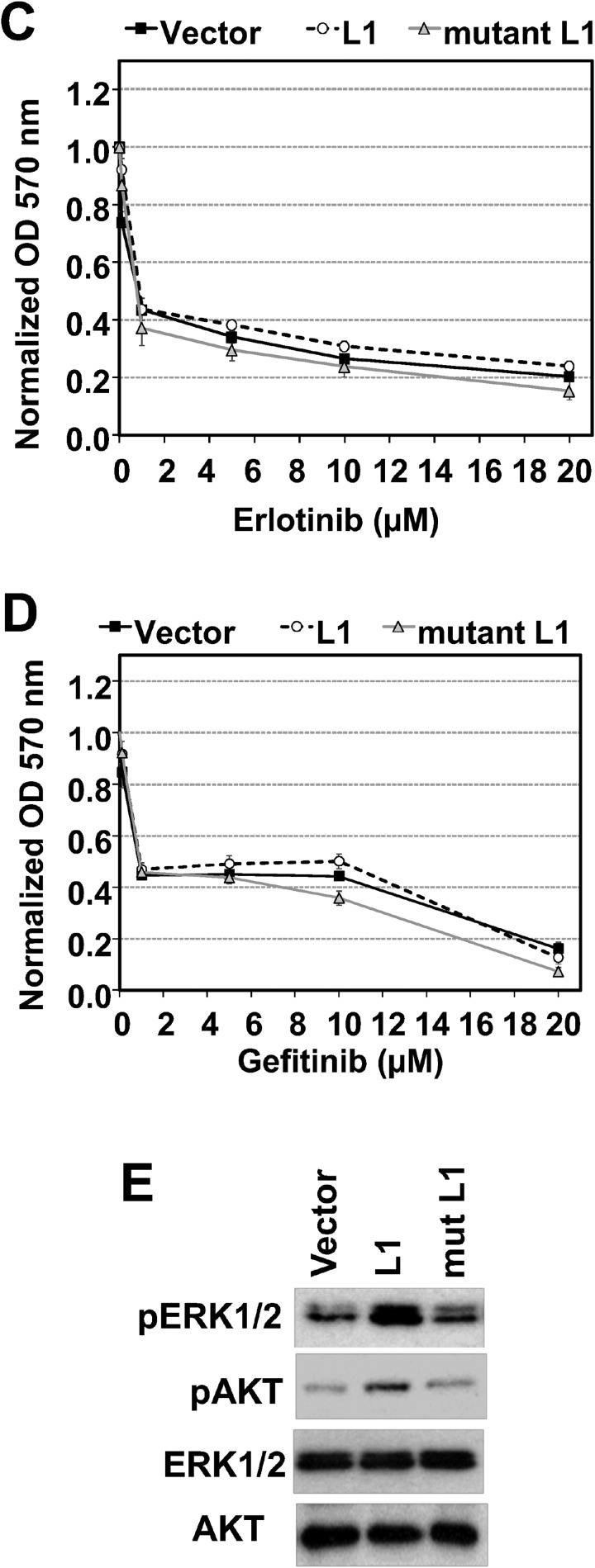
LINE-1 Modulates the Anti-proliferative Activity of TGF-β1 and Receptor Tyrosine Kinase Inhibitors Clones constitutively expressing wild type L1 (L1), a mutant L1 (mut L1), or empty vector were treated with various concentrations of TGF-β1 or control (**A**), Receptor tyrosine kinase inhibitors: sunitinib malate (VEGFR2, PDGFRβ and c-KIT inhibitor) (**B**), EGFR inhibitors- Erlotinib (**C**) and Gefitinib (**D**). After 72 h of treatment, proliferation was determined by the MTT assay and normalized to untreated or DMSO (vehicle for receptor tyrosine kinase inhibitors) for each cell type. Data represent the mean plus SEM for individual samples from three independent experiments. (**E**) Stably transfected cells were serum-starved for 24 h and cell lysates analyzed by immunoblotting for phospho-ERK1/2 (p-Erk1/2), phospho-AKT1 (p-Akt), ERK1/2 or AKT1 (Akt). Data are representative two independent experiments using clones #5 (wild type L1) and #13 (mutant L1).

Next, we determined if overexpression of LINE-1 is associated with the changes in cellular behavior characteristic of malignant phenotypes, such as resistance to anti-proliferative agents. TGF-β1 exerts dual functions with both tumor suppressor and tumor promoter activities, depending on cellular context and cross-regulation of growth factor signaling [11]. Stably transfected BEAS-2B clones expressing empty vector, wild type LINE-1 or mutant LINE-1 were challenged with varying concentrations of TGF-β1 for 72 h and cell proliferation measured based on cell counts or metabolic activity. All values were normalized to the proliferation rates of untreated cells for each individual clone. The anti-proliferative activity of TGF-β1 was decreased in clones expressing wild type or mutant LINE-1 compared to cells expressing empty vector (Figure 6A and Supplementary Figure 2). These data confirm the functional interactions between TGF-β1 and LINE-1 across multiple clones and indicate that retrotransposition competence is not required for this effect.

Because EMT promotes resistance to tyrosine kinase inhibitors during lung cancer progression, BEAS-2B cell clones were challenged with increasing concentrations of sunitinib maleate, erlotinib and gefitinib and proliferation rates examined. Sunitinib inhibits the tyrosine kinase activities of vascular endothelial growth factor receptor 2 (VEGFR2), platelet-derived growth factor receptor β (PDGFRβ), and c-KIT, while erlotinib and gefitinib inhibit EGFR. Clones constitutively expressing wild type and mutant LINE-1 were more resistant to the anti-proliferative actions of sunitinib malate than empty vector (Figure 6B). In sharp contrast, LINE-1 overexpression had no effect on the anti-proliferative activity of EGFR inhibitors (Figure 6C and Figure 6D). These data indicate that over expression of LINE-1 disrupts anti-proliferative activity of TGF-β1 and the tyrosine kinase inhibitor sunitinib in BEAS-2B cells, and this response is independent of reverse transcriptase activity.

Cancer cells can survive and acquire drug resistance by altering the expression and/or activation profiles of survival signaling pathways, including mitogen activated protein kinase (MAPK)/extracellular signal regulated kinase (ERK)1/2 and Akt [25, 26]. Moreover, ERK and AKT signaling contribute to acquired sunitinib resistance [27, 28]. To analyze whether LINE-1 overexpression affects these survival pathways, stably transfected BEAS-2B cells were serum starved for 24 hours and processed for immunoblotting analysis of these targets. Figure 6E shows increased phosphorylation of ERK1/2 in cells expressing wild type and mutant LINE-1 compared to empty vector. Cells expressing wild type LINE-1 also showed increased phosphorylation of AKT1 compared to either the mutant LINE-1 or empty vector (Figure 6E). These results indicate that LINE-1 overexpression is associated with prolonged activation of the MAPK (ERK1/2) and AKT1 survival pathways under serum depleted conditions, alterations that may contribute to drug resistance by upregulation of survival signaling pathways.

### LINE-1 induces oncogenic transformation of BEAS-2B cells

**Figure 7:**
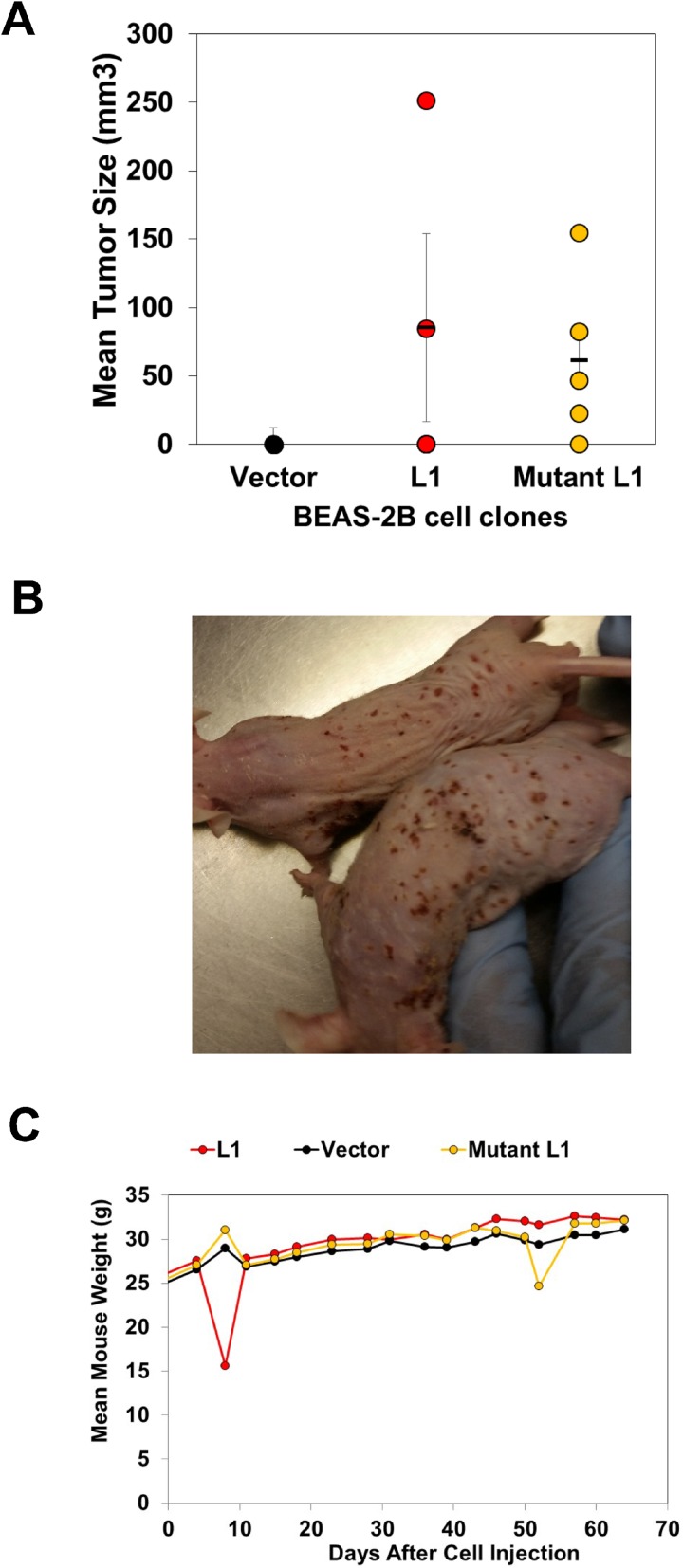
LINE-1 Induces Oncogenic Transformation of BEAS-2B cells Clones (1 X107 cells) constitutively expressing wild type LINE-1 (L1) (clone #5), a mutant L1 counterpart lacking reverse transcriptase activity (clone #13), or empty vector were mixed with matrigel and injected into 5-week old male Nu/Nu mice. (**A**) Tumor sizes 6-weeks after implantation. (**B**) Mice bearing cells expressing mutant L1. (**C**) Mouse weights.

Given the ability of LINE-1 to drive EMT programming and promote resistance to sunitinib maleate through modulation of tyrosine kinases, we next determined whether stable overexpression of LINE-1 promotes neoplastic transformation. To this end, athymic nude mice were implanted with BEAS-2B cell expressing LINE-1, mutant LINE-1 or empty vector (*n* = 5). Tumors were seen after six weeks in mice bearing cells that express LINE-1 (2/5), or mutant LINE-1 (4/5) (Figures 7A and 7B). No tumors developed in mice bearing empty vector cells. The tumors produced by cells expressing wild type LINE-1 showed maximum growth six-weeks post implantation, followed by lower growth rates and regression. In contrast, the tumors produced by cells expressing mutant LINE-1 continued to grow during the course of the experiment. Interestingly, these mice also developed skin ulcerations and strong inflammatory responses of the eyes bilaterally (Figure 7B), necessitating termination of the experiment. The regression of tumor growth in mice bearing wild type LINE-1 may be linked to genotoxicity of constitutively active LINE-1. No differences in body weights were seen in any of the treatment groups (Figure 6D). Together, these findings indicate that overexpression of LINE-1 induces oncogenic transformation in BEAS-2B cells, and this response is independent of reverse transcriptase activity and active cycles of retrotransposition.

## DISCUSSION

EMT exerts a profound influence on NSCLC progression, metastasis, and drug resistance [6–10], but the biological mechanisms involved are not well understood. Evidence is presented here that EMT programming in human lung epithelial cells couples the non-insertion activities of LINE-1 retrotransposon to the acquisition of oncogenic phenotypes. We propose the existence of a TGF-β1-LINE-1-EMT axis that functions in normal and transformed bronchial epithelial cells as a critical effector pathway that can be targeted for development of optimized therapies for patients with NSCLC.

TGF-β1 exerts dual functions serving as a tumor suppressor and tumor promoter depending on cellular context and cross-regulation of growth factor signaling [12]. While TGF-β1 induces anti-proliferative responses in epithelial, endothelial, neuronal and hematopoietic cells [2], its pro-tumorigenic activities are seen in cells undergoing EMT reprogramming to acquire motility and resistance to senescence and apoptosis [29]. In our studies, challenge of human bronchial epithelial cells with TGF-β1 or the lung carcinogen BaP activated EMT programming, and this response was associated with reactivation of endogenous LINE-1. However, TGF-β1 did not modulate LINE-1 retrotransposition events (data not shown), indicating that EMT reprogramming is effected via retrotransposition-independent mechanisms. This conclusion is in keeping with the ability of LINE-1 mutants unable to retrotranspose due to point mutations in reverse transcriptase to mediate EMT and induce tumors in nude mice. Of interest was the finding that LY2157299 completely blocked BaP-activated expression of LINE-1 mRNAs, thus specifying LINE-1 as a downstream effector of canonical TGFβ1 signaling during EMT reprogramming. This was confirmed in experiments showing that genetic knockdown of the downstream targets of TGF-β1 signaling, SMAD2 and SMAD3, also blocked LINE-1 induction. Interestingly, genetic knockdown of LINE-1 ORF1 did not inhibit TGF-β1-mediated reprogramming, showing that ORF1 protein may be necessary, but not sufficient to drive EMT programming in lung epithelial cells. We suggest that additional genes/proteins within the LINE-1 regulatory network participate in the EMT response and these relationships require directed investigation.

LINE-1 is a repetitive DNA sequence widely distributed throughout the human genome. Most copies of LINE-1 have been rendered inactive through 5′-truncation, with ∼100 full-length copies remaining in the genome able to mobilize upon epigenetic reactivation [30]. We have previously shown that epigenetic silencing of LINE-1 is effected via DNA methylation and recruitment of histone deacetylases through the repressive actions of E2F/RB complexes assembled on the LINE-1 promoter [18, 31, 32]. Given the ability of SMAD proteins to orchestrate chromatin remodeling [33], SMAD2 and SMAD3 may in fact participate in epigenetic control of LINE-1. The anti-proliferative activity of TGF-β1 was decreased in clones expressing wild type or mutant LINE-1, and this response may involve prolonged activation of the MAPK (ERK1/2) and AKT1 survival pathways. Thus, disruption of anti-proliferative control in lung epithelium by LINE-1 couples to phosphorylation-dependent regulatory pathways that promote carcinogenesis and cancer progression. AKT1 interferes with cytostatic SMAD signaling by sequestering SMAD3 away from TGF-βR-I and by activating mTOR kinase [34–36]. When transcribed as part of a larger transcript, LINE-1 may also regulate phosphorylation signaling by altering mRNA localization, changing mRNA stability and tuning the level of mRNA translation [37]. LINE-1 ORF1p is a substrate for several protein kinases that compete with adaptor proteins to disrupt kinase signaling [38, 39]. Overexpression of LINE-1 also promoted resistance to sunitinib, an inhibitor of the tyrosine kinase activities of vascular endothelial growth factor receptor 2 (VEGFR2), platelet-derived growth factor receptor β (PDGFRβ), and c-KIT. Cancer cells survive and acquire drug resistance by altering the expression and/or activation profiles of (MAPK)/ERK1/2 and Akt [25, 26].

The genome of lung cancer cells is one of the most frequently impacted by de novo LINE-1 insertions, with reports showing that > 50% of NSCLCs have increased LINE-1 ORF1 protein expression across a panel of different human lung neoplasms [20, 21]. Evidence is presented here that LINE-1 retrotransposon couples EMT programming with malignancy in human bronchial epithelial cells. LINE-1 modulates the expression of a large number of genetic targets involved in cancer progression and preferentially influences genes that regulate extracellular matrix biology, inflammation and cellular metabolism [16, 24]. Collectively, our findings identify a TGFβ1-LINE-1 axis as a critical effector pathway that can be targeted for the development of precision therapies during malignant progression of intractable NSCLCs.

## MATERIALS AND METHODS

### Materials

BaP was purchased from Ultra Scientific (Kingstown, RI). Recombinant human TGF-β1 was purchased from R&D Systems (Minneapolis, MN). Monoclonal anti-GAPDH, and horseradish peroxidase (HRP) linked anti-mouse IgG antibodies were from Santa Cruz Biotech (Dallas, TX). Rabbit anti-AhR (13790), anti-E-cadherin (24E10), anti-vimentin (D21H3), anti-N-cadherin (D4R1H), anti-ZO-1 (D7D12), anti-claudin-1 (8685), anti-Snail1 (C15D3), anti-Akt (C67E7), anti-phospho-Akt (T308) (C31E5E), anti-ERK1/2 (137F5), anti-phopho-ERK1/2 and horseradish peroxidase (HRP) linked anti-rabbit IgG antibodies were from Cell Signaling Technology (Beverly, MA). DMSO was from American Type Culture Collection (ATCC). Small interfering RNA (siRNA) duplex sequences were chemically synthesized and annealed by Thermo Fisher Scientific. The sequences of siRNA duplexes were 5′-CAGUUACUGUGGAAGGAAUtt-3′(TGFBR1 siRNA, Silencer^®^Select ID#s229438), (5′-GCUUCUCUGAACAAACCAGtt-3′ (SMAD2 siRNA, Silencer^®^Pre-designed ID#115715). 5′-GGCCCAGUGCAUAUGCAAUtt-3′ (SMAD3 siRNA, Silencer^®^Pre-designed ID#107877), 5′-CAAUGGAAGAUGAAAUGAAtt-3′ (ORF1 siRNA #1, Silencer^®^Select- Custom ID# s501620), 5′-GGGAGGACAUUCAAACCAAtt-3 (ORF1 siRNA #2 Silencer^®^Select- Custom ID# s501621), 5′-GGUGUGACUAACUAUGCAAtt-3′ (SNAIL siRNA #1, Hs_SNAI1_1 Qiagen), and 5 GAAUGUCCCUGCUCCACAAtt-3′ (SNAIL siRNA #2, Hs_SNAI1_5 Qiagen). BLAST analysis showed no homology to any sequence in the Human Genome Database, other than the intended target. The scrambled siRNAs used were Silencer^®^ Negative Control #1 siRNA (AM4635), Silencer^®^ Select Negative Control #2 siRNA (4390846), and Qiagen negative control (1022076). The siRNAs were transfected using Lipofectamine™ RNAiMAX (Thermo Fisher Scientific), according to the manufacturer’s directions.

### Polyclonal anti-human ORF1p antibody

A custom made, validated polyclonal antibody produced by New England Peptide LLC was diluted 1:1000 and used in all experiments.

### Cell culture and treatments

The human bronchial epithelial cell line BEAS-2B and Non-Small Cell Lung Cancer (NSCLC) cell lines (NCI-H460, NCI-H520 and NCI-H1993) were purchased from the American Type Culture Collection (ATCC). Cell lines were confirmed to be free of mycoplasma contamination (MycoAlert; Lonza). BEAS-2B were grown in LHC-9 medium while NSCLC cell lines were grown in RPMI media containing 10% FBS, Thermo Fisher Scientific, Grand Island, NY) in a humidified incubator at 37°C and 5% CO2. RPMI and LHC-9 medium were supplemented with 62.5 µg/mL penicillin and 100 µg/mL streptomycin (Thermo Fisher Scientific). Verification of all cell lines was performed by short tandem repeat (STR) using reference databases from ATCC (Genetics Core, University of Arizona, AZ). Cells were plated one day before treatments, and treated with desired concentrations of TGF-β1 (1–10 ng/mL) or BaP (0.1 µM-1 µM) as indicated in figure legends. For biochemical analyses, cells were lysed with buffer containing 150 mmol/L NaCl, 2 mmol/L EDTA, 50 mmol/L Tris-HCl, 0.25% deoxycholic acid, 1% IGEPAL CA-630 (pH 7.5), supplemented with protease and phosphatase inhibitor cocktails (EMD Millipore) for 5 min at 4°C, and then cleared by centrifugation at 16,000 × g for 10 minutes at 4°C. All protein concentrations were determined using the bicinchoninic acid assay (Thermo Fisher Scientific).

### RT-qPCR

Total RNA was isolated using the RNeasy Plus Kit (Qiagen) and 2 µg RNA digested with TurboDNase-I (Thermo Fisher Scientific). DNAse digested RNA (1 μg) was employed for cDNA synthesis using high-capacity cDNA reverse transcription Kit (Thermo Fisher Scientific). The resulting cDNAs (50 ng) were used as templates for RT-qPCR to analyze mRNA expression using *Power* SYBR^®^ Green PCR Master Mix and primers for L1-ORF1 (Forward: 5′-CCA AGTTGGAAAACACTCTGC-3′, Reverse: 5′-TGTGGCGTTCTCTGTATTTCC-3′), TGF-β1 (Forward: 5′-GGATACCAACTATTGCTTCAGCTCC-3′, Reverse: 5′-AGGCTCCAAATATAGGGGCAGGGTC-3′), and GAPDH (Forward: 5′- GATCATCAGCAATGCCTCCT-3′, Reverse: 5′- TGTGGTCATGAGTCCTTCCA-3). Fold changes were determined by comparing the ΔCT value of each product normalized to GAPDH as an internal control.

### Immunoblotting

Total cell lysates were resolved by SDS-Tris PAGE and transferred onto polyvinylidine fluoride membranes (Thermo Fisher Scientific) in Tris-glycine buffer containing 20% methanol. Proteins were detected by immunoblotting. Where indicated, membranes were stripped of bound antibodies using 62.5 mmol/L Tris-HCl (pH 6.7), 100 mmol/L 2-mercaptoethanol, and 2% SDS for 30 minutes at 60°C and reprobed as detailed in figure legends.

### Cell proliferation assays

Cell proliferation was examined directly counting cells or indirectly using a 3-(4,5-dimethylthiazol-2-yl)-2,5-diphenyltetrazolium bromide (MTT) assay [40]. The MTT assay monitors metabolic activity and is routinely used as an indirect measure of cell proliferation. Its utility was confirmed by cell counts and biochemical testing to rule out chemical interference or confounding by changes in cellular morphology. Briefly, 3,000 cells were seeded in quadruplicate in 96-well plates and allowed to adhere overnight. Cells were treated with different concentrations of TGF-β1 and incubated for different periods of time without changing the culture medium. The signal corresponding to medium with no cells was subtracted as background. Cell proliferation was determined by normalizing to the proliferation of untreated cells for each cell type.

### Stable cell lines

Expression vectors pB015^WT^ (wild type L1), pB016^MUT^ (L1 mutant carrying a single point mutation (D702Y) in ORF2 that destroys RT activity) and pB001^CTR^ (empty vector) generation has been previously described [16]. BEAS-2B cells were transfected with each expression vector using lipofectamine. Cells were incubated under standard conditions for three days before selection with hygromycin until the appearance of clones. Single clones were expanded and screened for L1 expression. Clones that showed similar expression of L1 and mutant L1 and no overt differences in cell growth under basal conditions were chosen for subsequent analyses.

### *In vivo* studies

The Institutional Animal Care and Use Committee (IACUC) at the University of Arizona approved all experimental procedures involving animals. Healthy male, weanling nude mice (Fox1nu) were purchased from Charles River Laboratories Inc. After acclimation for a week in the animal facility, mice were injected subcutaneously with a single cell suspension consisting of 10^7^ BEAS-2B cells expressing empty vector, L1 or mutant L1 (five mice for group) in 200 μL of matrigel into each flank. Tumor volume and body weight were recorded after every 2 or 3 days for 65 days.

### Statistical analysis

Experimental replicates were independent and performed on separate days. Comparisons were done between treated and control groups by ANOVA analysis as specified in figure legends.

## SUPPLEMENTARY MATERIALS FIGURES


