# Supplementary material for: LINE-1 couples EMT programming with acquisition of oncogenic phenotypes in human bronchial epithelial cells
Source: Oncotarget. 2017 Oct 23;8(61):103828–42. doi: 10.18632/oncotarget.21953 (PMC5732769; doi:10.18632/oncotarget.21953)
Supplement: Supplementary file 1 [file oncotarget-08-103828-s001.pdf]

# LINE-1 couples EMT programming with acquisition of oncogenic phenotypes in human bronchial epithelial cells

## SUPPLEMENTARY MATERIALS

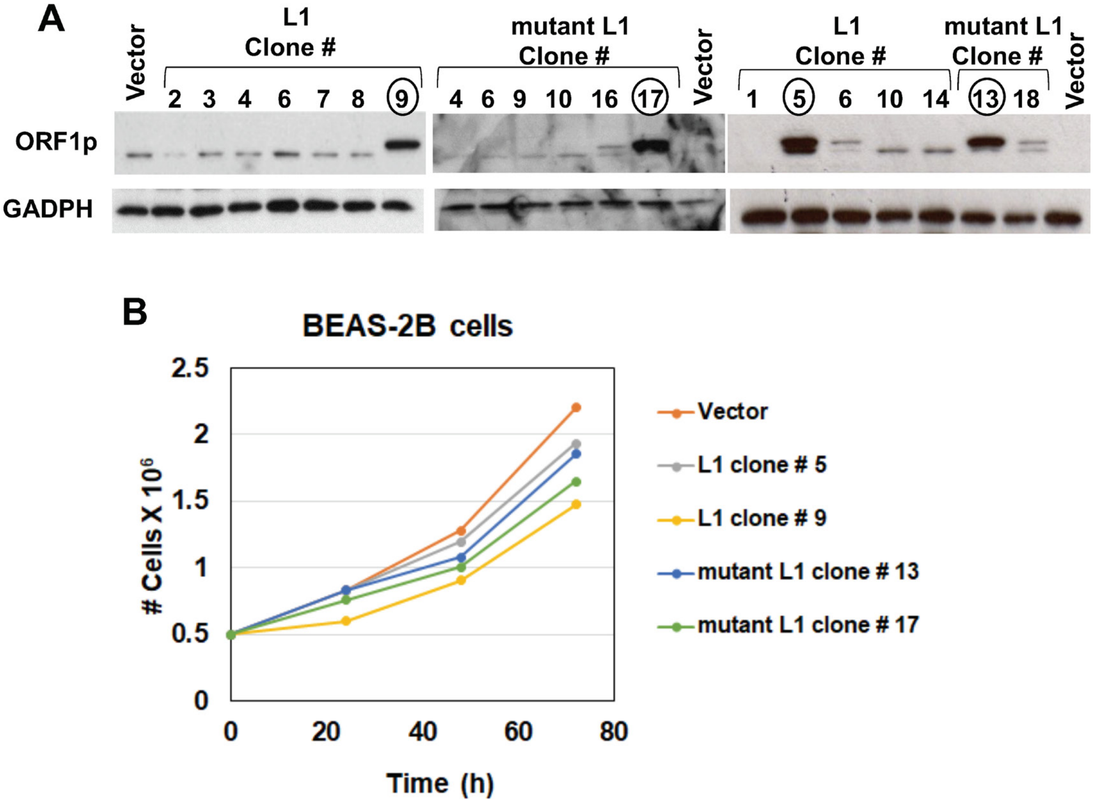


**Supplementary Figure 1: ORF1 protein expression and growth kinetics under basal conditions in BEAS-2B clones constitutively expressing wild type or mutant LINE-1.** BEAS-2B cells were stably transfected with wild type LINE-1 (L1); a mutant LINE-1 counterpart lacking reverse transcriptase activity (mutant L1) or empty vector and selected under pressure with hygromycin as described in Methods. (**A**) Immunoblotting of whole cell lysates from individual clones using antibodies against LINE1-ORF1protein or GAPDH as control. (**B**) BEAS-2B clones #5, 9, 13, and 17 (50,000 cells) were plated in 6-well plates at time 0 and growth kinetics monitored at 24, 48, and 72 hr. Cells were harvested and counted to quantify cell proliferation. Clones #5 and 9 represent cells transfected with wild type LINE-1, while clones # 13 and 17 represent cells transfected with mutant L1.

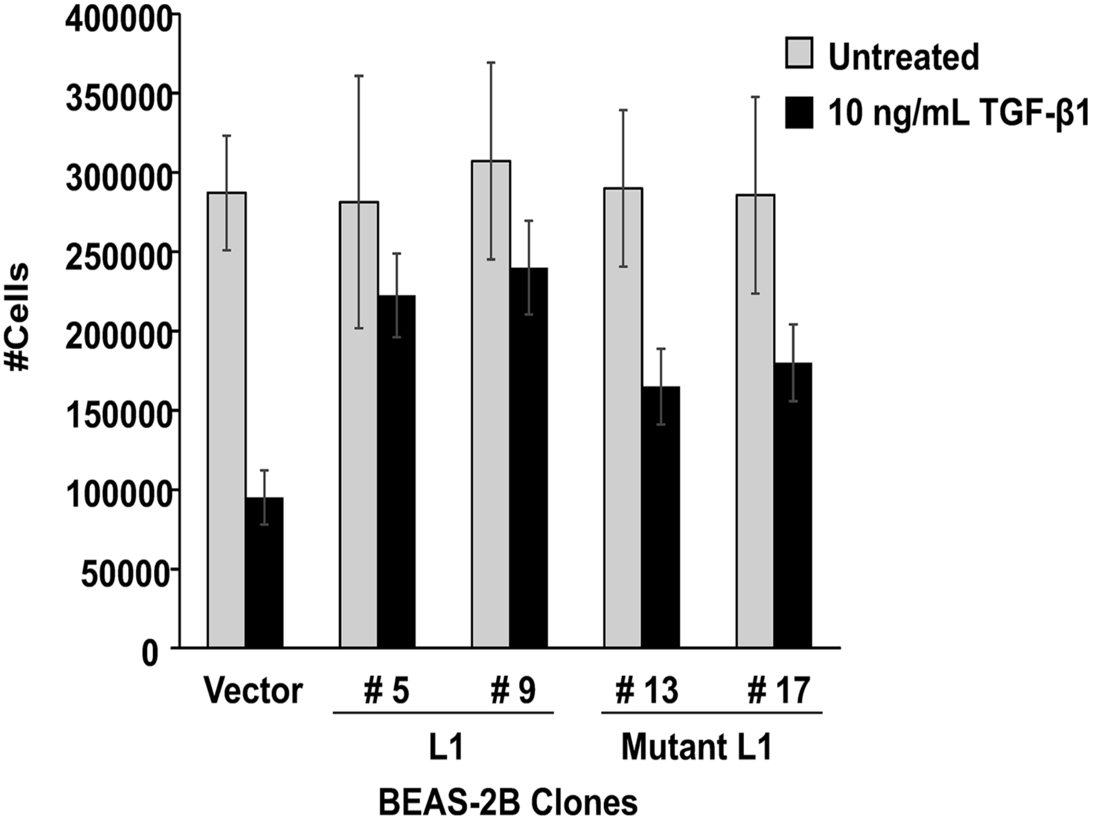


**Supplementary Figure 2: Inhibition of TGF-β1 Anti-proliferative Activity in BEAS-2B clones constitutively expressing wild type or mutant LINE-1.** Selected BEAS2-B clones (50,000 cells per replicate) were plated in 6-well plates one day before treatment. Cells were challenged directly with 10 ng/mL TGF-β1 and cell numbers measured after 72 hr. Clones #5 and 9 represent cells transfected with wild type LINE-1, while clones # 13 and 17 represent cells transfected with mutant L1. Proliferation was compared to clones transfected with empty vector.
